# Reactive Oxygen Ion Beam-Induced Deposition for Concurrent Purification of Platinum Nanostructures †

**DOI:** 10.3390/nano16040261

**Published:** 2026-02-17

**Authors:** Kyle Sprecker, Sujoy Ghosh, Philip D. Rack, Steven J. Randolph

**Affiliations:** 1 Department of Materials Science & Engineering, University of Tennessee, Knoxville, TN 37996, USA; ksprecke@utk.edu; 2 Center for Nanophase Materials Sciences, Oak Ridge National Laboratory, Oak Ridge, TN 37831, USA; ghoshs1@ornl.gov

**Keywords:** focused ion beam-induced deposition, platinum nanostructures, oxygen ion beam, direct-write nanofabrication, deposit purification, reactive ion–solid interactions

## Abstract

Oxygen-focused ion beam induced deposition (O-FIBID) enables the direct-write fabrication of Pt nanostructures while simultaneously enhancing purity concurrently through reactive oxygen–deposit interactions. By systematically varying the dwell time, accelerating voltage, and precursor pressure, the Pt content and conductivity can be controlled. Under optimum conditions, the Pt content reached 63 at.%. Across the dwell-time range used for resistivity measurements, the Pt content increased from 20 to 33 at.%, while the resistivity decreased from 2.9 × 10^4^ μΩ·cm to 1.2 × 10^3^ μΩ·cm, which is consistent with enhanced percolation through Pt grains and the lower intrinsic resistivity of the purer Pt deposit. The simulation results support a purification mechanism driven by the beam-induced activation of implanted oxygen balanced against the preferential sputtering of Pt. These results demonstrate O-FIBID as a viable method for the nanoscale direct write of conductive Pt without post-processing, and some deviations from conventional FIBID wisdom are observed. These results serve as a foundation for exploring nascent, reactive focused ion beam-induced deposition processes.

## 1. Introduction

Focused ion beam-induced deposition (FIBID) is a maskless, direct-write nanofabrication technique in which a focused ion beam decomposes adsorbed precursor molecules and results in the localized deposition of solid material [1,2,3]. The combination of ion beam imaging and direct surface modification within the same system has made FIBID a valuable tool in semiconductor processing [4]. It has been widely utilized for circuit editing [5], photomask repair [6], transmission electron microscope sample preparation [7], and the fabrication of three-dimensional nanostructures [8]. A key advantage of FIBID is the ability to deposit materials with tens of nanometer-scale precision without the need for conventional lithographic masks or resists [1].

During FIBID, a precursor gas is delivered locally through a gas injection system (GIS) and absorbs onto the substrate surface [3]. Subsequent ion irradiation induces precursor dissociation primarily through nuclear stopping mechanisms, especially at less than 30 keV [9]. The fragmentation of the precursor typically results in the desorption of volatile ligands, while other molecular fragments remain on the surface to form the deposit [10]. The process is spatially confined to the ion-solid interaction volume with lateral resolution determined by a combination of beam energy, ion species, dwell time, and precursor surface mobility [11,12,13]. For Ar^+^ and Xe^+^ ion sources, lateral feature sizes below 100 nm have been reported [1]; and for the He^+^ field ion microscope source, 16 nm has been achieved [14]. The localized nature of the interaction allows for site-specific deposition, which is particularly advantageous for device prototyping and nanoscale interconnect fabrication.

Platinum is among the most extensively studied materials for FIBID due to its high electrical conductivity, thermal stability, and chemical inertness [14,15,16,17]. These properties make Pt an attractive candidate for fabricating nanoscale electrical contacts, interconnects, and functional nanostructures in advanced device architectures. However, a major challenge is the incorporation of contaminants from the precursor, background gases in the chamber, and the ion beam [18,19]. In particular, carbon from the organometallic precursors commonly results in the deposits having an elevated resistivity and decreased metal content [20]. For a standard gallium ion source, Pt atomic fractions in deposits from the organometallic Pt precursor MeCpPtMe_3_ have been reported to be approximately 24 at.% Pt with the remainder consisting primarily of carbon [21]. For liquid gallium ion sources, the resistivity can also be modified by gallium implantation, whose concentration depends on the processing parameters [21]. As a result, Pt composition is widely used as a performance metric to evaluate the FIBID Pt structures [22].

To improve the metal content and reduce carbon contamination in Pt deposits for both focused ion and electron beam-induced deposition, a range of post-processing techniques have been developed, including electron beam curing [23,24,25,26,27], pulsed laser-assisted processes [28,29,30,31], and thermal annealing [32]. Electron beam curing relies on the interaction between electrons and reactive gases, like O_2_ or H_2_O, to dissociate and volatilize organic ligands that remain after precursor decomposition [23,26]. In these processes, the electron beam generates reactive species, such as oxygen radicals or hydroxyl groups, which oxidize carbon to CO or CO_2_ [32]. For laser-assisted deposition, an intermittent pulsed laser rapidly heats the growing deposit and thermally liberates the condensed carbon, where the duty cycle is low enough to not interfere significantly with the charged particle beam-induced growth [31]. Localized thermal annealing using either heated stages or focused laser irradiation has also been used to enhance the metal-to-carbon ratio by promoting organic ligand desorption and carbonization [29,33]. Beyond beam-mediated techniques, oxygen plasma exposure has been widely used to remove carbonaceous residues from nanostructures by converting carbon compounds into volatile species under reactive plasma conditions [34,35]. Additionally, preferential sputtering can affect the composition of depositions because lighter or less strongly bound species are removed at different rates than heavier or more strongly bound elements [36]. This phenomenon has been observed in GaN systems, where differences in sputtering yields lead to surface enrichment or the depletion of specific elements depending on ion energy and incidence [37]. Despite the many post-processing purification techniques, carbon impurities often remain in the FIBID deposited structures.

The composition and purity of platinum deposits produced by FIBID is highly sensitive to the ion beam parameters. Specifically, the ion type and the beam conditions play a crucial role in determining the deposit quality. For example, a study using Ga^+^ and Rb^+^ focused ion beams has shown that under identical deposition conditions, the Pt content was consistently lower for Ga^+^ FIBID deposits than for Rb^+^ deposits [15]. Moreover, ion beam current and accelerating voltage have been shown to greatly affect Pt deposition purity [38]. Higher beam currents increase the rate of precursor dissociation, which can affect the relative amount of carbon depending on the balance between ligand removal and precursor replenishment [39]. Furthermore, higher beam energies can cause competition between the deposition, sputtering, and implantation processes, which affects the overall composition of the deposit [14].

Unlike inert ion beams (He, Ne, Ar, Xe), oxygen ions introduce the possibility of chemically-assisted, concurrent purification during the deposition of Pt from the MeCpPtMe_3_ precursor. Previously, focused O^+^ ion beams have been used for milling diamond substrates, demonstrating simultaneous physical sputtering and chemical etching via oxidation [40,41]. Additionally, post-processing purification techniques have shown that reactive oxygen species can effectively break carbon bonds and facilitate CO/CO_2_ formation [42]. In particular, O_2_ plasmas exhibited higher carbon removal yields than fluorocarbon-based plasmas such as CF_4_, highlighting the potential for carbon removal during deposition [43].

Because deposition purity is dependent on both the ion beam species and beam parameters, this study investigates the use of an oxygen ion beam to deposit higher-purity Pt structures through tailored process parameters. The goal is for oxygen ions to chemically interact with the MeCpPtMe_3_ precursor and deposited material during decomposition to promote the formation of volatile by-products like CO or CO_2_. This oxidation mechanism could allow for the direct FIBID of high-purity platinum without requiring additional post-processing steps or the co-flow of additional precursor gases. Furthermore, the accelerating voltage, dwell time, refresh time, precursor pressure, and total ion dose are expected to play a role in the as-deposited purity by influencing the reaction dynamics, residence time of the intermediates, and the competition between deposition and sputtering. In this work, a focused oxygen ion beam is used to deposit platinum under various beam conditions to assess the feasibility and effectiveness of concurrent purification via what we will refer to as reactive oxygen FIBID or O-FIBID.

## 2. Materials and Methods

### 2.1. Experimental Deposition and Characterization Methods

Platinum depositions were performed on silicon substrates using a Thermo Fisher Scientific Helios 5 Hydra UX DualBeam plasma focused ion beam (PFIB) and scanning electron microscopy (SEM) system (Thermo Fisher Scientific, Hillsboro, OR, USA) equipped with a MultiChem gas injection system (GIS) (Thermo Fisher Scientific, Hillsboro, OR, USA). The precursor gas, trimethyl(methylcyclopentadienyl)platinum(IV) [MeCpPtMe_3_] (Thermo Fisher Scientific, Hillsboro, OR, USA), was introduced through the GIS nozzle positioned approximately 100 μm above the substrate. Local pressure was varied by controlling the duty cycle of the pulse width-modulated GIS valves. All other studied parameters were varied using the built-in patterning engine variables that will be discussed in detail subsequently. Long experimental runs were automated via Python scripting using an AutoScript application programming interface (API) (version 4.7, Thermo Fisher Scientific, Hillsboro, OR, USA; Python 3.5 embedded interpreter), allowing for unattended operation.

Given the duration of the experiments, all deposition conditions were performed multiple times and at a randomized time in order to ensure that temporal effects such as the system temperature, beam current, and pressure drift could be isolated if present. Post-deposition imaging and compositional analysis were performed in the PFIB system using secondary electron modes at a working distance of 4 mm. Energy-dispersive X-ray spectroscopy (EDS) was performed using an Oxford Instruments Ultim Max 170 detector (Oxford Instruments, Abingdon, UK), and each spectrum was collected for 30 s of live time to ensure consistency.

Electrical characterization was performed by depositing Pt lines over four microfabricated probe pad structures followed by I–V sweeps to measure resistance. A two-point probe configuration using a FormFactor Cascade CM300xi probe station (FormFactor Inc., Livermore, CA, USA) was used in conjunction with a Keithley 2450 SourceMeter (Tektronix, Inc., Beaverton, OR, USA) in voltage-control mode, sweeping from −0.1 V to +0.1 V in 0.002 V increments. At each voltage step, the resulting current was recorded after a brief stabilization delay (<100 ms). The resistance was extracted from the slope of the linear regression of the I–V curve. To determine the deposit resistivity, the dimensions of each line were determined via topographic imaging on an Asylum Research atomic force microscope (AFM) (Asylum Research, Santa Barbara, CA, USA; now part of Oxford Instruments).

### 2.2. Numerical Simulation of Pt IBID with Focused Oxygen Ion Beam

To investigate the primary factors influencing the observed deposition behavior and to help elucidate the role of the ion species, a numerical model was devised to track the evolving composition at a single pixel during the O-FIBID of Pt with a reactive oxygen beam. The cumulative processes that occur during ion beam-induced deposition conditions were handled by implementing single pixel dwell times, which were followed by a beam-off refresh period to mimic a scanned beam deposition where the pixel is “revisited” for each pass of the pattern. The essential elements of the model were the precursor surface coverage and beam-induced dissociation of the precursor into Pt and C species, sputtering, and the chemical reaction with oxygen. Beam-induced heating was assumed to occur and affect surface coverage dynamics among other processes that will be described in detail here.

In contrast to FEBID, FIBID must also consider ballistic processes due to the mass and velocity of the ion beam. Consequently, a competitive, parallel sputtering process was assumed to occur simultaneously with each element having a variable sputter yield. The model extends standard IBID frameworks by incorporating an additional reactive pathway for the ion-induced etching of carbon with the oxygen beam. There are several potential pathways by which volatilization could occur, but as a first step in understanding the complex dynamics, this model required simplification. To this end, it was assumed that oxygen must be shallowly implanted and energetically driven to form volatile CO_x_ species. The result would be a growing Pt_x_C_y_O_z_ deposit whereby incident ions could impact the near surface C_y_O_z_ and drive a volatilization reaction as a secondary carbon removal pathway.

**Figure 1:**
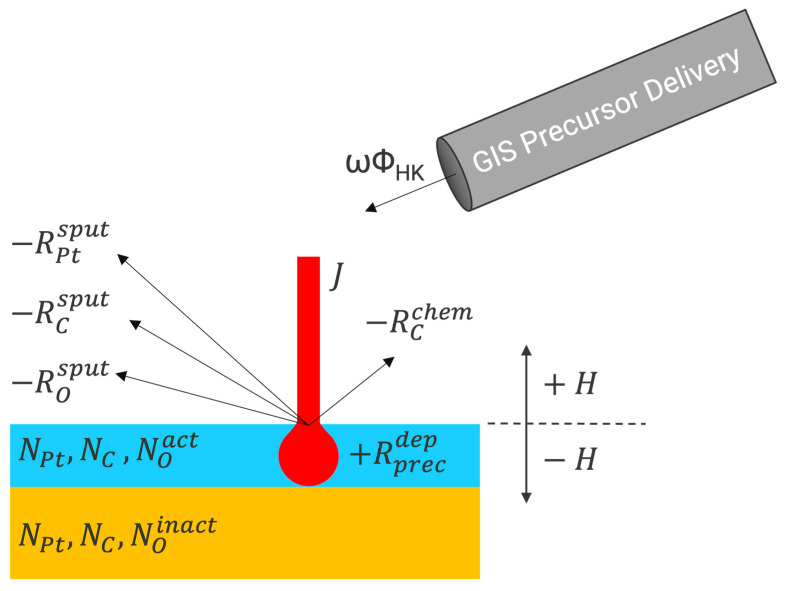
Graphical depiction of key concepts in the numerical model. The blue layer of the deposit is considered the active zone, where beam-induced oxidation of carbon can occur. The yellow layer is the inactive zone, where it is assumed the oxygen is trapped and no longer considered. The concentration of each element in the respective zones is labeled as well as the removal rates via sputtering and oxidation chemistry. GIS-enhanced precursor flux, *ωΦ_HK_*, and ion flux, *J*, drive the O-FIBID deposition rate. Surface growth and recession are denoted as *+H* and *−H*, respectively.

Since this process dynamically evolves during a growing deposition and the ion beam has limited penetration depth, a scheme was developed to bifurcate the available oxygen pool into active and inactive zones. The near-surface active zone was considered capable of producing the volatilization reaction, but more deeply buried oxygen was assumed to be trapped and inactive unless it could subsequently be moved to the active zone via a net removal process. In this manner, we would expect that conditions favoring high deposition rates would result in a greater likelihood of oxygen being trapped and not contributing to carbon removal, whereas low deposition rates should favor more near surface oxygen/beam interactions and more volatilization. Figure 1 is a simplified graphical schematic depicting the model and several of the most important concepts that it encompasses. Precursor flux, areal concentration changes of the primary species, additive and recessive deposit heights, and the bifurcation of oxygen into active and inactive zones are illustrated. Table S1 contains the key physical parameters, values, and units that were chosen for the numerical simulation.

Five coupled ordinary differential equations (ODEs) describe the system state in which platinum, carbon, oxygen areal densities, precursor surface coverage, and surface temperature were denoted by *N_Pt_*, *N_C_*, *N_O_*, *θ*, and *T_surf_*, respectively. To express film growth in physically intuitive terms, the total deposit thickness, *H* (in monolayers, ML), was related to the total areal density through an average ML areal density, *ρ_A_*, as

(1) $$H=\;\frac{N_{\mathrm{Pt}}+N_{C}+N_{O}}{\rho_{A}}.$$

Platinum accumulates through precursor dissociation and is removed through physical sputtering such that the net rate followed:

(2) $$\frac{dN_{Pt}}{dt}=R_{Pt}^{dep}-R_{Pt}^{sput}.$$

The rate of addition of component *i* via precursor breakdown was given by

(3) $$R_{i}^{dep}=\eta_{i}\;J\;\theta \eta_{prec}$$

where *i* is either Pt or C, *η_i_* is the number of atoms of species *i* (we assume constant 4:1 C:Pt ratios) of the precursor, *η_prec_* is the probability precursor dissociation, *J* is the incident ion flux, and *θ* is the ML precursor surface coverage governed by Langmuir adsorption kinetics.

The weighted sputter rates of component *j* were given by

(4) $$R_{j}^{sput}=J\;Y_{j}f_{j}$$

where *j* is Pt, C, or O. The sputter yield of component *j* is denoted *Y_j_*, and *f_j_* is the fractional composition of species *j* weighted by its collision cross-section with an oxygen ion. For the simulation results presented in this work, sputter yields of 0.08, 0.02, and 0.2 were assumed for Pt, C, and O, respectively, which are consistent with a mass dependent and momentum transfer efficiency-driven sputter process.

Carbon flux was assumed to have three competing pathways, including addition by precursor dissociation, removal by sputtering, and chemical loss, $R_{C}^{chem}$, via oxygen-mediated volatilization. We hypothesize that oxidation occurs only shallowly where the ion beam interacts with oxygen-implanted material; therefore, the film was partitioned into “active” and “inactive” zones. The active zone contains oxygen in a near-surface (active) layer accessible to the beam-induced “activation” of carbon—oxygen reaction. Deeper oxygen was treated as trapped and unavailable unless brought back to the surface via net erosion. The net carbon flux is therefore described by

(5) $$\frac{dN_{C}}{dt}=R_{C}^{dep}-R_{C}^{sput}-R_{C}^{chem}$$

The maximum supply rate of implanted oxygen species was first calculated as

(6) $$S_{O}=Jn_{O}\eta_{impl},$$

where *n_O_* is the number of oxygen atoms per ion (e.g., for O_2_^+^, *n_O_* = 2), and *η_impl_* is the fractional probability of incident oxygen ion implanting in the material, which we chose to be 75%. To ensure oxygen accumulation was realistically bounded, implantation was capped by a solubility-like limit, *f_O_*_,*max*_, and gated by the following function:

(7) $$g_{O}=\frac{f_{O}}{f_{O,max}}.$$

To describe oxygen implantation into the deposit, we implemented a dose-dependent functionality which mimics a system that increases its probability to absorb more oxygen species as beam damage accumulates. To this end, the maximum oxygen areal capacity that can be theoretically absorbed by the active layer was calculated as

(8) $$N_{O,max}=\rho_{A}\;{\;f}_{O,max}\;L_{act,eff}\;\left(1-e^{-{\beta (t)\sigma }_{defect}}\right),$$

where *β* is the ion dose applied at time *t*. The effective cross-section for defect site creation, *σ_defect_*, is a fitting parameter that controls how quickly during a beam dwell the oxygen capacity increases, which we chose to be 10^20^ s^−1^.

A deficiency to the ODE approach to this problem is the lack of any spatial variation in composition, which is sacrificed to gain computational throughput for these qualitative simulations. To approach reality where oxygen is only implanted to a certain depth and chemistry and sputtering can only occur in the near-surface region, we bifurcated the deposit into activity zones by using Equation (1) to estimate the thickness of the deposit and assume a change in behavior centered around a defined depth within the deposit. The active depth over which implantation and oxidation chemistry occurs was assumed to follow the form:

(9) $$L_{act,\;eff}=L_{act}\tanh\left(\frac{H}{L_{act}}\right),$$

where *L_act,eff_* is the effective depth over which the oxygen–carbon chemistry and sputtering is active, and *L_act_* is a constant and assumed to be 5 ML. This functional form is purely a convenient means to ensure a smooth transition between the initial period of growth and latter growth stages that asymptotically approaches a maximum when H approaches *L_act_*. This effectively renders that oxygen buried more deeply than *L_act_* is unable to participate in volatilization chemistry.

While *N_O,max_* defines the time dependence of the film’s ability to retain accept oxygen, we presume that the amount of oxygen absorbed into the film is only a fraction of the oxygen delivered (*S_O_*). Thus, we define the effective oxygen implantation rate, *S_eff_*:

(10) $$S_{eff}=S_{O}{\left(1+{\left(\frac{N_{O}}{N_{O,max}}\right)}^{m_{ret}}\right)}^{-1},$$

where *m_ret_* is a mathematical construct to smooth the transition between efficient oxygen uptake, and when excess oxygen is simply discarded (pumped away), as the capacity becomes throttled as *N_O_* approaches *N_O,max_* asymptotically.

Using the above relationships, the rate of carbon removal through chemistry can then be described by:

(11) $$R_{C}^{chem}=Jk_{chem}f_{C}g_{O},$$

with the rate constant for CO_x_ formation, *k_chem_*, following Arrhenius kinetics as

(12) $$k_{chem}=k_{chem}^{0}e^{\frac{-E_{chem}}{k_{B}T_{surf}}},$$

where $E_{chem}$ is the activation energy of the oxidation reaction, and *k_B_* is the Boltzmann constant, and $k_{chem}^{0}$= 500 was chosen as a rate constant prefactor fitting parameter. After incorporating the rate of thermally activated oxygen diffusion out of the active layer, $R_{O}^{diff}$, the oxygen areal rate equation becomes

(13) $$\frac{{dN}_{O}}{dt}=S_{eff}-\left(R_{O}^{diff}+R_{O}^{sput}+n_{O}R_{C}^{chem}\right).$$

Precursor coverage was modeled as a temperature-dependent Langmuir adsorption process up to a single monolayer. The rate equation for precursor surface coverage, *θ*, was described by the rate equation:

(14) $$\frac{d\theta }{dt}=\;R_{ads}-\;\left(R_{des}^{thermal}+R_{cons}\right),$$

where $R_{ads}$ is the adsorption flux of precursor molecules, $R_{des}^{thermal}$ is the thermal desorption rate of the precursor, and $R_{cons}$ is the rate of precursor consumption through deposition.

The thermal desorption rate constant was assumed to follow Arrhenius kinetics:

(15) $$k_{des}=k_{des}^{o}\;e^{\frac{-E_{des}}{k_{B}T_{surf}}},$$

where $k_{des}^{o}$ is the desorption rate prefactor, and $E_{des}$ is the activation energy for precursor desorption such that

(16) $$R_{des}^{thermal}=k_{des}\theta .$$

The effective sticking coefficient of precursor was assumed to follow

(17) $$S_{eff}\left(T\right)=S_{0}e^{\frac{-E_{ads}}{k_{B}}\left(\frac{1}{T_{surf}}-\frac{1}{T_{amb}}\right)},$$

where $S_{0}$ is the base sticking coefficient (assumed to be 5 × 10^−3^), and $E_{ads}$ is the activation barrier for adsorption. Values from Fowlkes et al. [44] were used the simulations. The temperature-dependent sticking coefficient is then used to calculate the instantaneous precursor adsorption rate, *R_ads_*:

(18) $$R_{ads}\left(T\right)=\frac{{\omega \phi }_{HK}\left(1-\theta \right)S_{eff}}{N_{s}},$$

where $\phi_{HK}$ is the Hertz–Knudsen gas flux, which is determined by the gas pressure, and *ω* is a local gas enhancement factor due to the GIS needle [45], which we assume to be an order of magnitude consistent with previous reports on our system [46] at 1 × 10^3^. Ion-induced desorption was assumed to be a separate process and governed by the product of a fixed probability for desorption, the ion flux, and the precursor coverage.

Given that several processes such as precursor adsorption, the sticking coefficient, carbon–oxygen reaction rates, and oxygen out-diffusion are all temperature-dependent processes, a very simple beam-induced time-dependent heating model was implemented by estimating the ion-induced power density as

(19) $$q^{\prime \prime }=JE_{beam}e^{-}$$

where $e^{-}$ is the elementary charge, and $E_{beam}$ is the beam energy in eV. The steady-state surface temperature, $T_{\infty }$, when the beam is on was calculated as

(20) $$T_{\infty }=\left(T_{amb}+q^{\prime \prime }R_{th}\right),$$

where $R_{th}$ is the effective thermal resistance of the deposit, and $T_{amb}$ is the ambient temperature of 298 K. The heating is only operative during the dwell, and the pixel is assumed to instantly return to $T_{amb}$ during the refresh period. The surface temperature is then calculated during beam dwell by solving

(21) $$\tau_{th}\frac{dT_{surf}}{dt}=T_{\infty }-T_{surf},$$

where $\tau_{th}$ is a thermal time constant. To a first approximation, we set the thermal resistance such that the maximum surface temperature at steady state for our 2.1 nA beam condition was approximately 365 K with the steady state being reached in approximately 3.5 μs. This modest temperature increase is a reasonable assumption given that the beam-induced heating of bulk materials is far less than in dimensionally constrained structures [47,48] such as pillars and lamellae, and the time constant chosen for heating is on the order expected [49] for beam-induced heating. As an example, Figure S2 shows an example of the thermal model output for a single 50 μs dwell.

The above series of equations represents the core physics and chemistry required to recreate the model of Pt O-FIBID. The model was subsequently numerically solved as a system of simultaneous ordinary differential equations. Each of the chemical and physical processes were sufficiently modularized to allow for an investigation of the importance of each of these processes independently. The large number of tuning parameters and physical constants in the model ensures that the model can only be used to qualitatively assess the deposition process. However, the ability to enable/disable physics modules allowed for a focus on one physical process at a time, which provided an understanding of the relative importance of the processes. The results of these analyses are found in the Supplementary Information.

## 3. Results and Discussion

### 3.1. Beam and Gas Dynamics Effects During Pt O-FIBID

**Figure 2:**
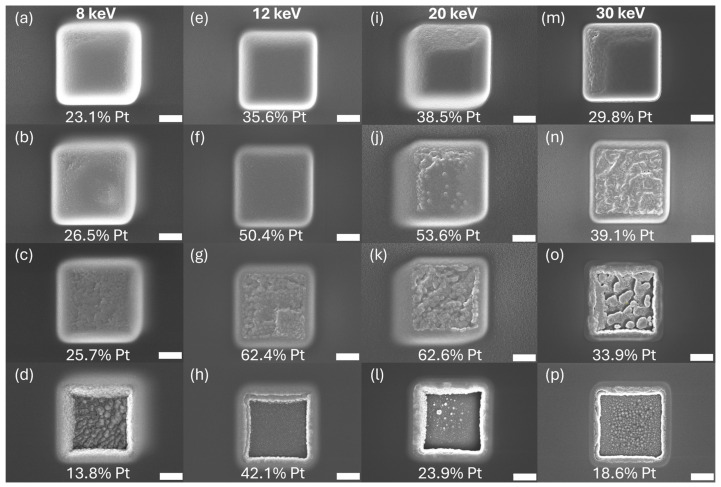
SEM images of O-FIBID Pt depositions and corresponding average Pt composition (at.%) for 8 keV (**a**–**d**), 12 keV (**e**–**h**), 20 keV (**i**–**l**), and 30 keV (**m**–**p**) with varying dwell times: (**a**) 5 µs, (**b**) 15 µs, (**c**) 25 µs, (**d**) 100 µs, (**e**) 0.5 µs, (**f**) 10 µs, (**g**) 30 µs, (**h**) 100 µs, (**i**) 0.5 µs, (**j**) 10 µs, (**k**) 23 µs, (**l**) 100 µs, (**m**) 1 µs, (**n**) 15 µs, (**o**) 35 µs, and (**p**) 150 µs. All scale bars are 1 μm in length.

**Figure 3:**
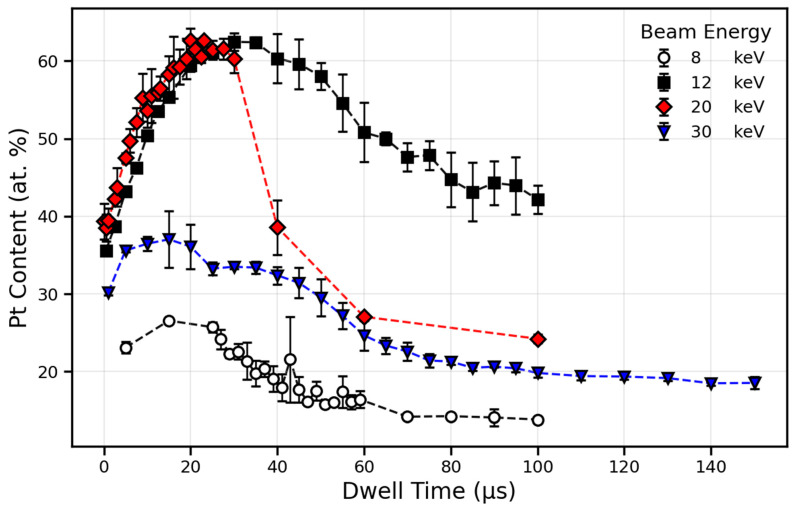
Averaged Pt composition (at.%) versus dwell time for 8, 12, 20, and 30 kV with error bars of ±1 standard deviation.

**Figure 4:**
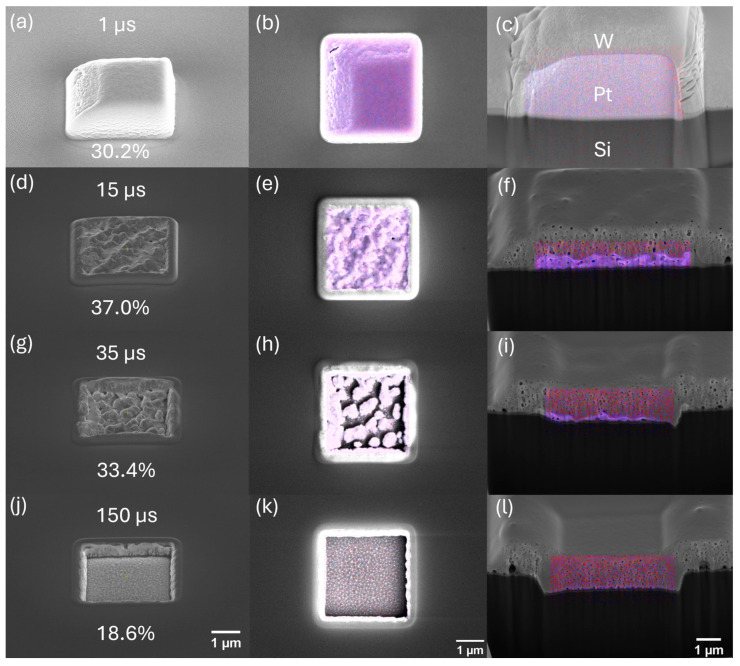
SEM micrographs and cross-sectional images with an overlaid EDS of O-FIBID Pt deposits synthesized at (**a**–**c**) 1 μs, (**d**–**f**) 15 μs, (**g**–**i**) 35 μs, and (**j**–**l**) 150 μs dwell times at 30 kV beam energy, 0.23 nA beam current, 1% duty cycle, and 10 min deposition time with a tungsten capping layer deposited prior to cross-sectioning. Pt and C correspond to blue and red, respectively. Images (**b**,**e**,**h**,**k**) have no tilt angle, and the rest were taken at a tilt angle of 52 degrees.

We hypothesized that the oxygen-assisted volatilization of carbon during deposition would be primarily driven through implanted oxygen being activated by subsequent irradiation. As such, we sought out conditions that would result in high oxygen accumulation such as during precursor-depleted deposition. To test this, a study was performed to map out a parameter space where the transition between precursor depletion occurred. A series of depositions with varying dwell times and beam energies was carried out, followed by the SEM imaging of morphology, and EDS compositional analysis, which were both acquired at an accelerating voltage of 10 kV with a probe current of 1.6 nA. To further drive precursor depletion, a 1% duty cycle for the MultiChem GIS was used (default Pt duty cycle is >80%). Features were patterned as 3 × 3 μm^2^ square boxes with a 10 μm spacing between adjacent depositions. A 50% pixel overlap and dynamic all-direction scan strategy were empirically determined to provide the most consistent and uniform deposition. Each deposition was carried out for 10 min. The oxygen ion beam was varied at beam energies of 8, 12, 20, and 30 keV. Corresponding beam currents were maintained as near 0.23 nA as possible with inherent variations due to column optics. For each beam energy, a range of dwell times were investigated: 8 keV (5–100 μs), 12 keV (0.5–100 μs), 20 keV (0.1–100 μs), and 30 keV (1–150 μs). The difference in dwell time ranges was necessary given the finding that the precursor depletion dwell time window was dependent on the beam energy. The SEM images of the wide variability in Pt morphologies are shown in Figure 2, while the composition measurements are plotted in Figure 3. Cross sections and EDS mapping are provided in Figure 4. 

Due to the thin nature of the deposited features and the dependence of electron penetration depth on local deposit thickness, EDS spectra were acquired individually for each deposition. The contribution from the silicon substrate was not consistent across all measurements with the Si peak appearing in some spectra and not in others depending on deposit thickness and morphology. To ensure consistency in the compositional analysis, the silicon substrate peak was excluded from quantification. While an EDS analysis of thin, topographically varying deposits is inherently non-ideal, this approach represents the most practical method available for assessing the deposition composition.

Across all beam energies, the Pt composition increased with dwell time, reached a maximum, and then decreased at longer dwell times with accompanying surface morphological changes (Figure 2 and Figure 3). Interestingly, the maximum platinum content and the optimal dwell time both showed a strong dependence on beam energy with 12 keV and 20 keV showing the highest average platinum content. The origin and nature of the beam energy dependence are the subject of ongoing study, and a detailed analysis is beyond the scope of this work. Instead, we focus here on understanding kinetic effects such as dwell time and precursor pressure.

It was anticipated that an increased dwell time would increase the Pt content as in the shorter dwell regions of Figure 4, but the peak and decay were more surprising. We presume that this trend is attributed to several competing factors: precursor replenishment, ion-stimulated dissociation, by-product desorption, sputtering, and the beam-induced oxidation of carbonaceous fragments. At short dwell times, the surface never becomes fully depleted of precursor molecules, resulting in an efficient deposition and/or incomplete ion–precursor dissociation. At the shortest dwell times, the growth rate is high and results in implanted oxygen being trapped subsurface and unable to assist in volatilization. This yields smooth and laterally uniform deposits with good morphology indicative of a deposition-dominated regime but low-to-moderate Pt content (Figure 2a,d,g,j).

As the dwell time increased to intermediate values, the depositions shifted toward more precursor depleted regimes, resulting in lower deposition rates and improved accessibility for the carbon in the deposit to access the subsurface implanted oxygen. Through this mechanism, the Pt content increased due to the simultaneous carbon removal through oxygen-assisted chemistry and differential sputtering, causing surface roughening and grain coarsening. The highest average Pt purities were achieved in this region, reaching 62.4 at.% at 30 μs for 12 keV and 62.6 at.% at 23 μs for 20 keV (Figure 2g,k). The roughened surface morphologies observed at intermediate dwell times are indicative of a transition from an efficient, deposition-dominated regime to a sputtering/chemistry-dominated regime in a manner reminiscent of post-deposition coarsening [29] during the purification of Pt deposits.

At the longest dwell times, precursor depletion effects begin to dominate, and the platinum concentration surpasses its maximum and begins to decline as shown in Figure 4. Here, we speculate that the competitive effects of oxidation chemistry and differential sputtering begin to shift their relative contributions. It is known that sputter yields can increase with increasing dwell time [50] during ion milling processes. A decrease in the relative contribution of oxygen chemistry coupled with increasing sputter yields may be responsible for a shift in mechanism from chemistry-dominated to differential-sputtering-dominated. The differential species removal of a heterogeneous deposit can certainly lead to surface roughening (Figure 2c,g,k,o) and void formation (Figure 2d,h,l,p). The large Pt grains in the deposition interior for intermediate dwell times of Figure 2 are consistent with an efficient chemical removal of carbon being more favorable than differential sputtering that would favor carbon enrichment due to the presumed higher sputter yield of platinum [51] compared to carbon. Eventually, as the dwell time is further increased, an increase in the overall sputter yield could potentially overtake the carbon/oxygen reaction rate, resulting in carbon enrichment. To truly understand the nature of this regime transition, more studies were necessary to track the oxygen content of the deposit to search for evidence of this shift in balance between oxygen-assisted carbon etching and differential sputtering.

As evidenced by Figure 2, platinum composition clearly is not the only metric of optimal deposition. The morphology of the deposit must also be considered for suitability to a particular application. To gain a more thorough understanding of the morphological effects in each kinetic regime, we cross-sectioned and performed the SEM imaging of four representative deposits in Figure 4. These are cross-sectional image deposits from the 30 keV beam energy, 0.23 nA beam current, and 1% duty cycle experiment. These images further support the trends observed in the dwell study where the film’s thickness and density transition from beam-limited, low Pt content into a regime where sputter and chemistry are well balanced and produce optimal Pt content. At the shortest dwell time of 1 μs (Figure 4a,b,c), the deposit forms a continuous layer 2.46 μm thick with small, scattered Pt grains. Increasing the dwell time to 15 μs (Figure 4d,e,f) results in a thinner deposit of 500 nm with larger Pt grains and the highest platinum content. At 35 μs dwell time (Figure 4g,h,i), the deposit thickness decreases to 251 nm, and the film becomes irregular with larger grains and extensive void formation as the Pt content decreases. Additionally, the extensive interfacial smearing, grain coarsening, and void formation observed in this regime are consistent with high-dose ion mixing and competitive deposition–sputtering processes previously reported for ion-irradiated metallic systems [52]. By 150 μs dwell time (Figure 4j,k,l), negligible Pt remains with no measurable thickness. As a result, it is important to balance the need for compositional purity and the thickness/uniformity of deposition. In use cases such as a protective layer for sample preparation, uniformity may be of greater importance. For applications such as circuit editing and making electrical contacts, purity may be the more important property, as it is the primary contributor to resistivity. Individual element distributions are shown in Figure S6.

**Figure 5:**
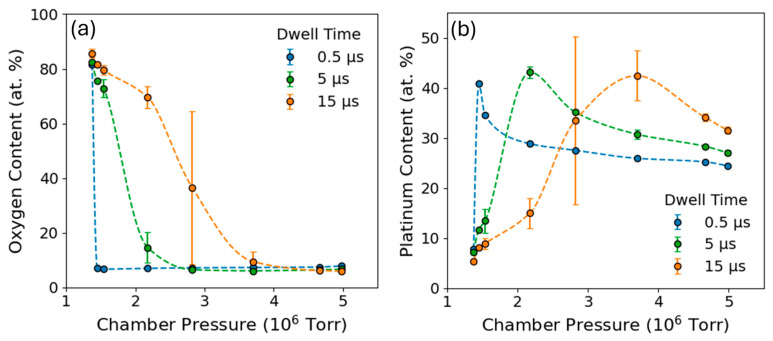
Average oxygen (**a**) and platinum (**b**) composition as a function of chamber pressure at dwell times of 0.5 μs, 5 μs, and 15 μs.

An additional study was performed to alter precursor depletion by varying the precursor pressure with a fixed beam energy at 30 keV. An advantage to adjusting precursor depletion through pressure is that it removes the coupling between dwell time and loop time (refresh time) during deposition, since for a given dwell time, the pixel refresh is identical for all pressures. The disadvantage is the limited window of pressure control, which is about one order of magnitude as opposed to many orders of magnitude of control when adjusting the dwell time. For completeness, we performed pressure series at a range of dwell times. These experiments were conducted at a beam energy of 30 keV with a beam current of 2.44 nA. Depositions were patterned as 10 × 10 μm^2^ square pads on silicon with 15 μm spacing between adjacent features, and each deposition was carried out for 3 min under continuous rastering. The precursor flux was systematically varied by adjusting the duty cycle to 0.1%, 0.5%, 1%, 5%, 10%, 20%, 50%, and 95% in combination with dwell times of 150 ns, 0.5 μs, 1 μs, 5 μs, 10 μs, 15 μs, and 20 μs for each duty cycle. Triplicate depositions were performed for each condition, and the average of the oxygen and platinum compositions are plotted in Figure 5 as a function of chamber pressure. For clarity of the plots, only the 0.5, 5, and 15 μs data are presented in Figure 5, since they convey the overall trends effectively. Figure S2 contains the full pressure studies at seven different dwell times. Figure S5 provides top–down SEM micrographs for 0.15, 1, 10, and 20 μs dwell times for each of the duty cycles, while Table S2 and Table S3 contain the corresponding average Pt content and average pressures, respectively.

The first notable observation from these experiments was the large variation in oxygen content as a function of chamber pressure (Figure 5a). Such a large range of oxygen concentrations in the deposits further reinforces the idea that for a full understanding of O-FIBID, a better understanding of oxygen behavior is necessary. Looking specifically at low chamber pressures, it must be considered that the overall deposition rate is low due to precursor depletion, and we suspect that competitive sputter and carbon etching allow oxygen to accumulate more readily in the slowly evolving deposit volume. It is important to note that at these precursor-depleted conditions, we cannot rule out that a portion of the oxygen increase could also be attributed to oxygen retention in the silicon substrate given its affinity for oxygen. As the chamber pressure is increased, deposition rates increase, and there is a reduced likelihood of interaction with the substrate. In these cases, as oxygen necessarily becomes distributed throughout a larger volume of material, the oxygen naturally becomes more diluted. Also consistent with a volumetric deposition rate-controlled oxygen concentration is that for increasing dwell times, higher pressures can still support higher oxygen concentrations.

However, as seen in Figure 5b, the platinum content behavior implies more complexity than an overall deposition rate-controlled process. We observe that for a given dwell time, there is a specific pressure at which the platinum content is highest. The increase in platinum content is accompanied by a sharp decay in oxygen content, which we view as too abrupt to be fully explained by oxygen dilution. We assume this is due to carbon and oxygen loss through volatilization.

**Figure 6:**
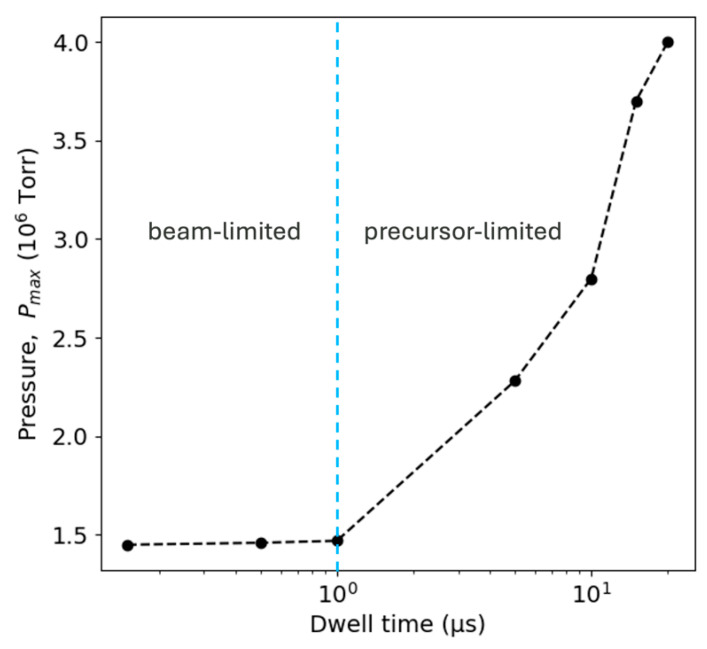
Plot of the experimentally determined pressure/dwell time combination resulting in maximum platinum content in Pt O-FIBID deposits.

The efficiency of this platinum purification process is ultimately governed by the combination of pressure and dwell time for a given set of beam conditions. When we analyze the pressure study results for the pressure–dwell combination that results in the most efficient platinum purification (maxima of all curves in Figure S2), we find that as dwell times increased, the pressure required to reach the maximum Pt content also increased, as shown in Figure 6. The beam-limited regime is characterized by short dwell times that have little effect on the pressure, *P_max_*, at which platinum content is maximized. Conversely, in the precursor-limited, long-dwell regimes, the precursor pressure becomes very consequential to the maximization of platinum content.

From the combination of qualitative imaging and these kinetic experiments, we propose a model of O-FIBID of platinum that is a complex interplay of precursor coverage, differential sputtering, and the oxidation and removal of CO_x_ characterized by three distinct regimes:

(i) A low-pressure regime in which precursor depletion results in high oxygen implantation and retention. Here, deposition rates are very low, which means that there is little carbon to be removed chemically, and the preferential sputtering of Pt results in low Pt content.
(ii) An intermediate pressure regime where precursor replenishment is sufficient to sustain a loss of carbon and oxygen through beam-induced volatilization. Here, the chemical process and preferential sputter are well balanced and result in the maximum platinum content.
(iii) A high-pressure deposition regime where abundant precursor coverage promotes fast growth, but oxygen becomes highly deficient and can no longer support effective volatilization. Here, preferential sputtering of platinum again begins to dominate the process and results in reduced platinum content.

To assess the ability to control the composition and electrical properties of O-FIBID, we also performed a series of wire depositions to measure the resistance and calculate the resistivity as a function of deposition conditions guided by the previous kinetic experiments. The Pt depositions were performed at an accelerating voltage of 12 kV and a 1.5 nA beam current on an SiO_2_ substrate pre-patterned with isolated electrical contacts. The deposition geometry was a 20 × 5 μm rectangular box with 50% pixel overlap in both the x and y directions. Each feature was deposited for 5 min, and dwell times of 0.25, 1, 5, 10, 15, and 20 μs were selected to span the compositional range observed in the previous Pt deposits at 12 kV. Because the dwell time can be adjusted by several orders of magnitude, varying the dwell time was chosen for the resistivity study instead of varying the precursor duty cycle.

**Figure 7:**
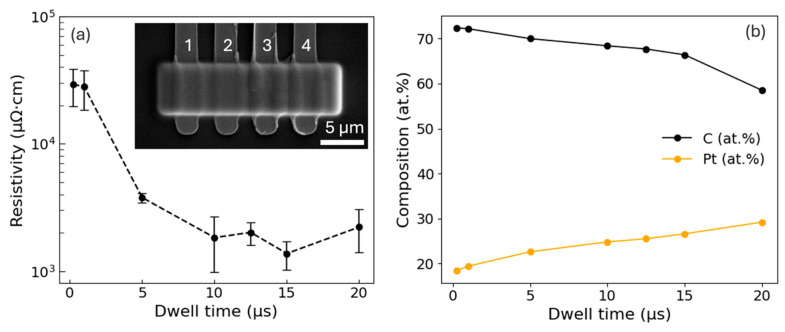
(**a**) Resistivity of O-FIBID Pt deposits versus dwell time from 0.25–20 μs, showing an initial decrease in resistivity and eventually stabilizing at higher dwell times; inset: representative SEM micrograph of four-probe structures used for measurement. (**b**) Platinum and carbon composition of the O-FIBID Pt deposits in (**a**).

Following deposition, current–voltage measurements (Figure 7) were collected across adjacent pads (1–2, 2–3, and 3–4, Figure 7 inset) to measure the resistance of each section of the Pt deposits. The resulting I–V data were linear and symmetric around 0 V. The resistance was extracted from the slope of the linear regression of the I–V curve, and the resistivity was then calculated from the cross-sectional area and the distance between the contacts. The deposit width and length were determined from the designed pattern geometry, while the thickness was obtained from AFM topographic imaging. All resistivity measurements were performed under ambient laboratory conditions and are the average of three replicate measurements per dwell time condition. The results are plotted in Figure 7a, which shows that resistivity decreases by more than an order of magnitude with increasing dwell time—from roughly 2.9 × 10^4^ μΩ·cm at 0.25 μs to 1.2 × 10^3^ μΩ·cm at 10 μs. The resistivity plateaus at values near 1–2 × 10^3^ μΩ·cm, which is consistent with the reduced sensitivity of Pt content at longer dwell times (Figure 3 and Figure 4b). This reduction in resistivity is likely caused by both the increasing metallic content and more effective percolation through interconnected, larger Pt grains.

Figure 7b also shows the compositional variation in these deposits, which are positively correlated with resistivity measurements. In these deposits, the platinum content increases with dwell time from approximately 20 at.% at 0.25 μs to 33 at.% at 20 μs, while the carbon content decreases from 80 at.% to 67 at.%. Interestingly, the average Pt content for these resistivity measurements is lower than predicted by the earlier voltage–dwell and pressure–dwell experiments. This difference may be attributed to reduced precursor adsorption on the SiO_2_ substrate compared to Si. The MeCpPtMe_3_ precursor has a weaker surface affinity on oxides, which leads to faster desorption and reduced precursor availability [53]. In addition, the insulating nature of SiO_2_ can result in surface charge accumulation altering the local electric field and further reducing the precursor residence time. Also, local charging on the insulating substrate can effectively defocus the beam, leading to lower current density, which also impacts FIBID kinetics. While the explanation for the difference in composition is not fully understood, it is not surprising that the substrate could have a substantial impact. However, because of the correlation between Pt content and resistivity, improving the Pt purity on the SiO_2_ substrate should be possible given more optimization experiments.

While the decrease in resistivity with increasing dwell time correlates with increased Pt content and improved metallic percolation, additional factors may also contribute to the resulting electrical properties of the deposits. For example, variations in microvoid density [54], local compositional inhomogeneities [10], or ion-induced migration and the redistribution of carbon, oxygen, and other residual species [55] could influence charge transport pathways. Although these effects were not independently quantified in this work, they may contribute to the observed resistivity trends and merit further investigation.

These resistivity values tend to lie between those of Pt as-deposited by Ga^+^ FIBID and FEBID. In low-density, impure FEBID Pt deposits, resistivity values range from 10^4^ to 10^7^ μΩ·cm [32], whereas Ga^+^ FIBID ranges from 10^2^ to 10^5^ μΩ·cm [56]. Even though higher Pt content seems to be achievable with O-FIBID, the gallium incorporation during Ga^+^ FIBID can yield lower resistivity [56] at the expense of doping the material with mobile gallium atoms. While FIBID Pt can be purified via post-processing strategies [57] or laser annealing [10], O-FIBID incorporates a reactive oxygen beam for concurrent oxidation and volatilization to reduce impurity and improve resistivity, which provides several obvious benefits for nanoscale fabrication and warrants further study.

### 3.2. Numerical Simulation of Pt O-FIBID and the Role of Reactive Oxygen

To better understand the primary effects that the oxygen beam has on Pt O-FIBID, a series of continuum simulations were performed using the model described in Section 2.2. A wide array of physical parameters are available for model tuning, and we made our best attempt at selecting physically meaningful values with key parameters being listed in Section 2. One of the more impactful parameters, which can significantly alter the balance between the chemical oxidation of carbon and physical sputtering, are the effective sputter yields of the individual deposit components. Sputter yields for individual species, while oversimplified, were selected based on relative sputter yield ratios or Ar^+^ collisions with the relevant species [51], as these values were the closest in mass to the O^+^ beam. The absolute values of the sputter yields (a scalar product of these ratios) was used to help tune the model to reasonable deposition rates observed experimentally. From these reports, the absolute sputter yields used in the simulation were fixed at 0.2/0.08/0.02 for O/Pt/C. For brevity and convenience, the remainder of the physical parameters in the model are listed in Supplementary Table S1, where we focus on the qualitative behavior of the simulations.

**Figure 8:**
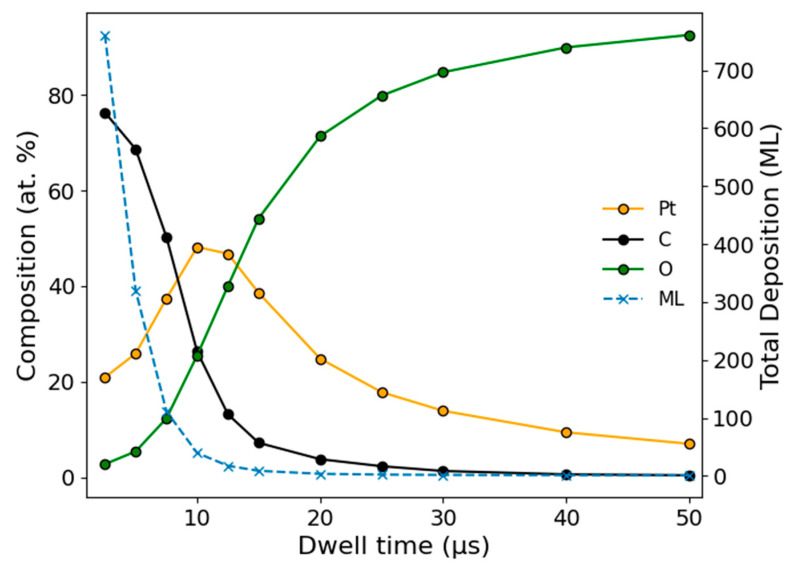
Simulated study of dwell time effects on deposition thickness and elemental composition for 30 keV, 2.2 nA, and 180 s deposition at a chamber pressure of 3.6 × 10^−6^ torr.

The first set of simulations was performed using a 30 keV, 2.2 nA nominal beam current over a 10 × 10 μm box to mimic experimental parameters. The nominal beam diameter of 250 nm was determined from the optics model of the DualBeam, and a beam overlap of 50% was used to calculate the total pattern loop time, assuming a 1 ms settle time per loop. For a 180 s deposition simulation, the number of loops is determined by the total time divided by the product of the dwell time and the number of pixels plus the fixed beam settle time. A set of 180 s simulations for a fixed chamber pressure of 3.6 × 10^−6^ torr was performed as a function of dwell time, and the areal composition of Pt, C, and O was tracked in addition to the estimated deposit thickness. These results are plotted in Figure 8, which displays a sharp decrease in the overall deposition thickness over the range of dwell times from 1 to 50 μs, which agrees with experimental observations. Importantly, the model also predicts an initial increase in Pt content from 20% to nearly 50% when the dwell time approaches 10 μs, after which a slow decay in Pt content is observed. This is also consistent with the observations presented in Figure 5. Thus, the model qualitatively supports our hypothesis of a beam-activated implanted oxygen-driven purification process in the more beam-limited, short dwell regimes. In more gas-depleted regimes, the preferential sputtering of Pt becomes dominant and begins to decrease the relative Pt content while also resulting in lower deposition rates and higher residual oxygen in the deposit.

**Figure 9:**
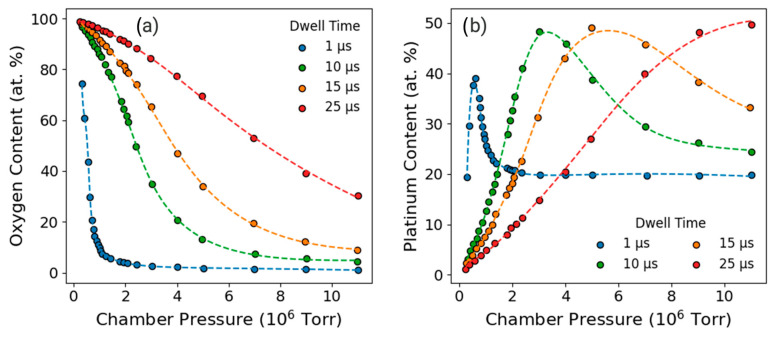
Series of simulations of the (**a**) oxygen and (**b**) platinum content of 180 s depositions carried out at 30 keV, 2.2 nA as a function of chamber pressure.

While these initial simulations were promising, for completeness, we used the same model parameters to evaluate the effects of changing the precursor pressure in combination with dwell time as in the experiments of Figure 5. Here, we simulated four different dwell times with a variation in chamber pressure over an order of magnitude. Again, the assumptions of the model with implanted oxygen driving carbon volatilization prove to qualitatively describe the experimental observations quite well. Comparing the experiments in Figure 5 with the results of simulations in Figure 9a, we see that the model adequately predicts the rapid oxygen depletion with chamber pressure at short dwell times with the decay being less sensitive to pressure at longer dwell times. Figure 9b shows that rapid oxygen depletion is correlated to a rapid increase in Pt content, but once oxygen is depleted in the film, Pt purification is arrested, and the preferential sputtering of Pt results in a reduction in Pt content.

To truly evaluate the individual effects of the physical and chemical process and confirm that the model indeed supports the implanted oxygen model, it was necessary to perform a sensitivity analysis. This allowed a much deeper understanding of the behavior of the model when various physics were enabled or disabled independently, allowing a clearer interpretation of the Pt O-FIBID process. Figure S3a displays the oxygen and platinum content variation as a function of chamber pressure with and without beam heating. When beam heating, chemistry, and sputtering are all active (solid lines), the results resemble those of Figure 8. As beam heating is limited in our bulk substrate case to a few tens of degrees Celsius, the effects are not dramatic but do indeed have an effect. When beam heating is disabled (dashed lines), the maximum Pt content is lower and shifted to lower chamber pressures. Oxygen is more rapidly depleted in the non-beam heating case. We interpret this as showing that higher deposition rates without heating lead to a slightly higher oxygen content for a given pressure than when beam heating is used. Additionally, when heating is enabled, the etch reaction rate increases, in turn reducing the carbon available, leading to an oxygen excess for the same chamber pressure.

Figure S3b contains another analysis, with beam heating enabled, but observing the effects of disabling either the oxidation chemistry or sputter removal components independently. Here, we observe a very broad non-monotonic behavior for the chemistry-only case, whereas the sputter-only case shows a monotonic increase in Pt with pressure. The sputter-only case also highlights another important point in that the overall oxygen content is lower in the sputter-only case. Even though oxygen loss through CO formation is not present, the highly efficient sputtering of oxygen is the dominant factor in oxygen concentration. Therefore, the absence of sputtering in the chemistry-only case allows for artificially elevated implanted oxygen levels, which combined with the absence of a Pt removal pathway explains the higher Pt content compared to when all physics are enabled.

The next analysis was focused on determining the model sensitivity to changes in the relative sputter yields of Pt and C. As an extreme case, we performed similar sweeps of pressure and a range of dwell times by inverting the Pt to C sputter yields so that the C sputter yield is 4x that of Pt. Figure S4 shows a series of pressure sweeps as in Figure 9 with the only difference being the inverted sputter yields. While the maximum Pt content shifts to higher values in this case, the non-monotonic behavior of Pt is preserved. Additionally, the effects of dwell time are similar in that the maximum Pt content is achieved at higher pressures as the dwell time is increased.

These sensitivity analyses provide more confidence that the balance between the sputtering of Pt, C, O, and the beam-activated oxidation of carbon are driving the sharply non-monotonic Pt behavior we observe experimentally and in simulation. These results have significant practical impact on how reactive Pt O-FIBID must be viewed going forward. To begin, it is a highly complex process requiring a delicate balance between the activation of implanted oxygen and the preferential sputtering of Pt. From a practical standpoint, the default patterning dwell time values that are designed to limit gas depletion result in depositions in a very narrow process window for controlling the Pt content. In general, both the simulation and experiment show much more process latitude when operating at high chamber pressures and what are normally considered excessive dwell times. Default FIBID pattering values are usually in the sub-1 μs regime, which is likely due to historical bias from Ga^+^ FIBID. Here, we show both through experimental and qualitative simulation that optimal deposition (prioritizing Pt content) from O-FIBID occurs at dwell times normally associated with extreme sputtering conditions. Together, our results and simulations indicate that for cases featuring high-sputter-yield metals such as Pt and likely Au, a limit to oxidative chemistry from the oxygen beam exists, which is not overcome by simply pushing farther toward gas depletion. While lower-sputter-yield metals such as Ti and W may be more amenable to carbon reduction due to their sputter resistance, they are also susceptible to oxidation, which will likely lead to metal oxide formation and additional complexity.

## 4. Conclusions

This study demonstrates that O-FIBID can purify platinum deposits concurrently with direct write deposition. The interaction between reactive oxygen ions, the MeCpPtMe_3_ precursor, and the growing deposit yields several competing processes (deposition, sputtering, and oxidation) that can be tuned through adjusting beam parameters (beam dwell time, accelerating voltage, and precursor pressure) to achieve high-purity Pt deposits without post-processing steps. The results reveal that deposition purity and electrical performance are governed by the balance between precursor availability, ion-driven reactions with implanted oxygen, and the preferential sputtering of platinum.

At short dwell times and low pressures, a beam-driven carbon reaction with implanted oxygen dominates the reduction in carbon content in the deposits. Prolonged dwell times result in gas depletion, the preferential sputtering of platinum, high residual oxygen content, low deposition rates, and gradually reducing Pt content. Increasing the chamber pressure and dwell times result in higher Pt purity over a larger pressure/dwell time process window. Optimal dwell time values that prioritize Pt purity are more than an order of magnitude higher than the default patterning files suggest.

Electrical measurements confirm that increased Pt content correlates with improved conductivity. The as-deposited resistivities, reaching approximately 1.2 × 10^3^ μΩ·cm, are comparable to conventional Ga^+^ FIBID and are significantly lower than as-deposited FEBID Pt films. These results demonstrate that the reactive oxygen ion beam can act as an effective deposition and purification mechanism.

O-FIBID provides a promising pathway for the direct, maskless fabrication of conductive platinum nanostructures with reduced impurity content and without the need for post-deposition treatment. The optimization of precursor flux and beam parameters can enhance deposition purity and highlights the broader applicability of this approach to other metal–organic precursors and reactive ion chemistries.

## Data Availability

Data is contained within the article or Supplementary Material.

## Supplementary Materials

The following supporting information can be downloaded at https://www.mdpi.com/article/10.3390/nano16040261/s1, Table S1: Parameter values for numerical simulations in Section 3.2. Figure S1: Simulated temperature profile for a 50 μs dwell using a 30 keV beam, 2.2 nA oxygen ion beam 250 nm in diameter. For simulation results, it should be noted that only dwell times less than approximately 3 μs do not reach the steady state value, *T_∞_*. This dwell time temperature trace is from the final loop of a 31 loop exposure simulation, but all loops have an identical temporal profile. Figure S2: Full data set from experiments presented selectively for clarity in Figure 5. Shown here is the (a) oxygen content and (b) platinum content vs. chamber pressure for 7 different dwell time conditions. Figure S3: Sensitivity analysis on the numerical simulation with (a) all physics enabled, and beam heating disabled, and with (b) chemical etching and physical sputtering alone enabled. Legends indicate which of the 3 main physics modules are operative. Only platinum and oxygen content are shown for clarity as carbon is the remaining balance. Figure S4: Sensitivity analysis of the numerical simulation with sputter yields of Pt and C inverted from those used throughout the work. Sputter yields used here were Pt = 0.08 and C = 0.02. Three pressure sweeps at dwell times of: (a) 500 ns, (b) 10 μs, and (c) 20 μs. Maximum Pt values are higher in this case, but the general non-monotonic behavior is still observed. Figure S5: Top-down SEM micrographs of 10 × 10 μm square Pt deposits fabricated using a beam energy of 30 keV and beam current of 2.44 nA. Precursor depletion was systematically varied by adjusting the precursor duty cycle (a–d) 0.1, (e–h) 0.5, (i–l) 1, (m–p) 5, (q–t) 10, (u–x) 20, (aa–dd) 50, and (ee–hh) 95%. Within each duty-cycle group, the dwell time increases from top to bottom, corresponding to 0.15, 1, 10, and 20 µs, respectively. The scale bars are identical between images. Table S2: Average platinum composition as a function of dwell time and duty cycle, reported as mean ± one standard deviation from triplicate depositions. Table S3: Corresponding average precursor pressure (chamber pressure minus base pressure) and chamber pressures associated with each duty cycle. Figure S6: Cross-sectional SEM and EDS analysis of Pt deposits fabricated at 30 kV usingdwell times of (a) 1 µs, (b) 15 µs, (c) 35 µs, and (d) 150 µs. Panels (a–d) show cross-sectional SEM images overlaid with the combined EDS elemental maps. Corresponding individual elemental distributions are shown to the right for each dwell time: carbon (red: a1–d1), oxygen (green: a2–d2), tungsten (purple: a3–d3), and platinum (blue: a4–d4).

## Abbreviations

The following abbreviations are used in this manuscript:

FIB	Focused ion beam
FIBID	Focused ion beam-induced deposition
FEBID	Focused electron beam-induced deposition
O-FIBID	Oxygen focused ion beam-induced deposition
GIS	Gas injection system
Pt	Platinum
Ga^+^	Gallium ion
O^+^	Oxygen ion
at.%	Atomic percent
SEM	Scanning electron microscopy
EDS	Energy-dispersive X-ray spectroscopy
AFM	Atomic force microscopy
SRIM	Stopping and Range of Ions in Matter
TRIM	Transport of Ions in Matter
