# Supplementary material for: Reactive Oxygen Ion Beam-Induced Deposition for Concurrent Purification of Platinum Nanostructures
Source: Nanomaterials (Basel). 2026 Feb 17;16(4):261. doi: 10.3390/nano16040261 (PMC12943533; doi:10.3390/nano16040261)
Supplement: Supplementary file 1 [file nanomaterials-16-00261-s001.zip › nanomaterials-4125878-supplementary.pdf]

Supplementary Materials

Reactive Oxygen Ion Beam Induced Deposition for Concurrent Purification of Platinum Nanostructures

Kyle Sprecker [1], Sujoy Ghosh [2], Steven J. Randolph [2*], and Philip D. Rack [1,2*]

[1] Department of Materials Science & Engineering, University of Tennessee, Knoxville, TN 37996, USA

[2] Center for Nanophase Materials Sciences, Oak Ridge National Laboratory, Oak Ridge, TN 37831, USA

[*]Correspondence: randolphsj@ornl.gov and prack@utk.edu

Parameter	Default	Units	Parameter	Default	Units
**Scan Timing and Environment**			**Surface Properties**		
Dwell time per pixel	15×10^{-6}	s	Molecular site density	1.27×10^{18}	$m^{-2}\cdot ML^{-1}$
Total deposition time	180	s	Atomic site density	1×10^{19}	$m^{-2}\cdot ML^{-1}$
Chamber pressure	1×10^{-6}	Torr	Maximum precursor coverage	1	ML
Ion beam energy	30	keV	Precursor efficiency prefactor	10	–
Beam diameter	250×10^{-9}	m	Initial film thickness	0.1	ML
Beam current	2.2×10^{-9}	A	Initial oxygen atomic fraction	0	–
Pixel overlap factor	0.5	–	**Physical Sputtering**		
Write-field size	10×10^{-6}	m	Pt sputter scaling factor	0.2	–
Per-loop settle time	1×10^{-3}	s	C sputter scaling factor	0.05	–
Extra beam-off refresh time	0	s	O sputter scaling factor	0.5	–
Ambient temperature	298	K	**Deposition Stoichiometry**		
**Precursor Coverage**			Pt atoms per dissociation	1	atoms
Sticking coefficient (reference)	0.005	–	C atoms per dissociation (base)	4	atoms
Desorption time constant (reference)	12.5×10^{-3}	s	Maximum C atoms per dissociation	4	atoms
Desorption prefactor (Arrhenius)	1×10^{9}	s^{-1}	**Chemical C/O Etching**		
Desorption activation energy	0.01	eV	Chemical etch activation energy	0.2	eV
GIS enhancement factor	1000	–	Active oxygen layer depth	20	ML
Adsorption activation energy	0.05	eV	Oxygen consumed per C removed	1	$O\cdot C^{-1}$
**Beam Heating**			Oxygen implanted per ion	2	$O\cdot ion^{-1}$
Thermal Conductivity	10	$W\cdot m^{-1}\cdot K^{-1}$	Implantation efficiency	0.75	–
Mass density	1000	$kg\cdot m^{-3}$	Chemical etch prefactor	2000	$C\cdot ion^{-1}$
Heat capacity	100	$J\cdot kg^{-1}\cdot K^{-1}$	Oxygen diffusion prefactor	1×10^{4}	s^{-1}
Thermal conduction length	0.5×10^{-6}	m	Diffusion activation (E/kB)	400	K
Thermal solver speedup factor	5000	–	Maximum active oxygen fraction	0.6	–
			Oxygen retention sharpness	3	–
			Oxygen reaction order	1	–

Table S1: Parameter values for numerical simulations in Section 3.2

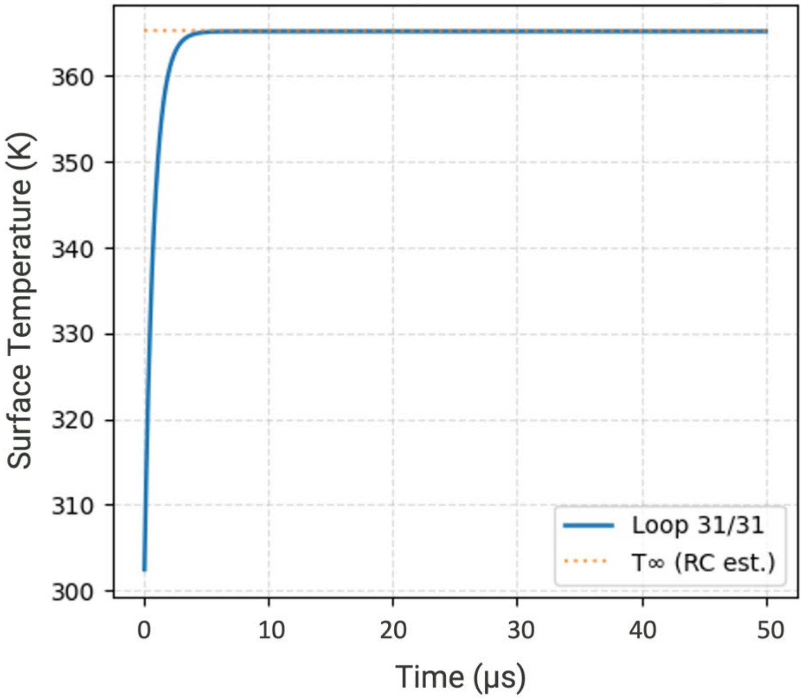

Figure S1: Simulated temperature profile for a 50 µs dwell using a 30 keV beam, 2.2 nA oxygen ion beam 250 nm in diameter. For simulation results, it should be noted that only dwell times less than approximately 3 µs do not reach the steady state value, T_{∞}. This dwell time temperature trace is from the final loop of a 31 loop exposure simulation, but all loops have an identical temporal profile.

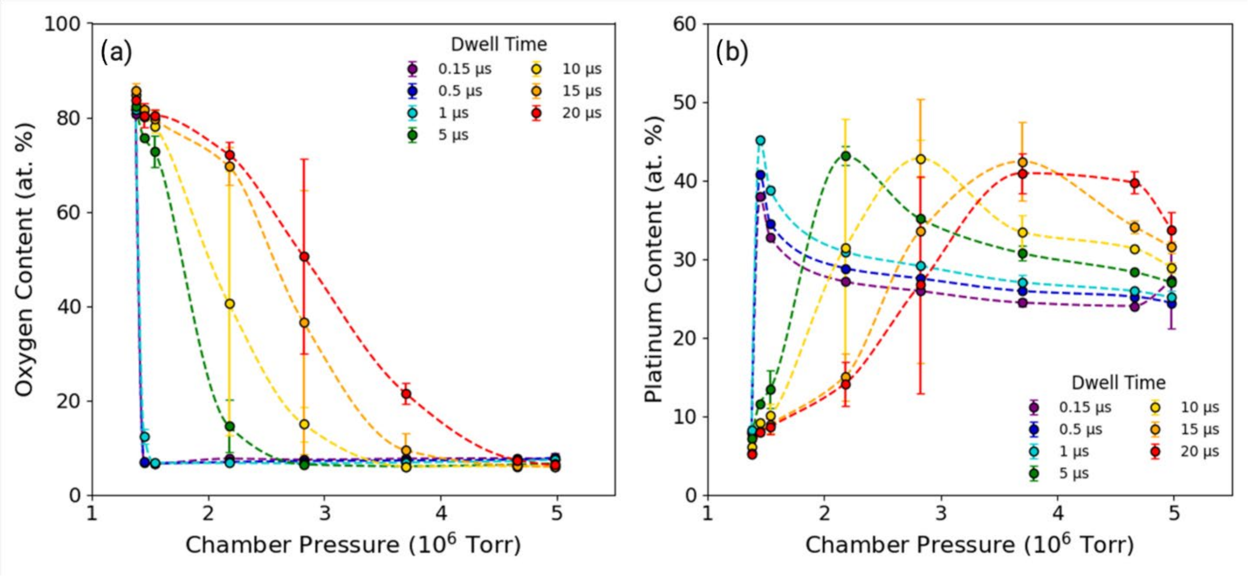

Figure S2: Full data set from experiments presented selectively for clarity in Figure 5. Shown here is the (a) oxygen content and (b) platinum content vs. chamber pressure for 7 different dwell time conditions.

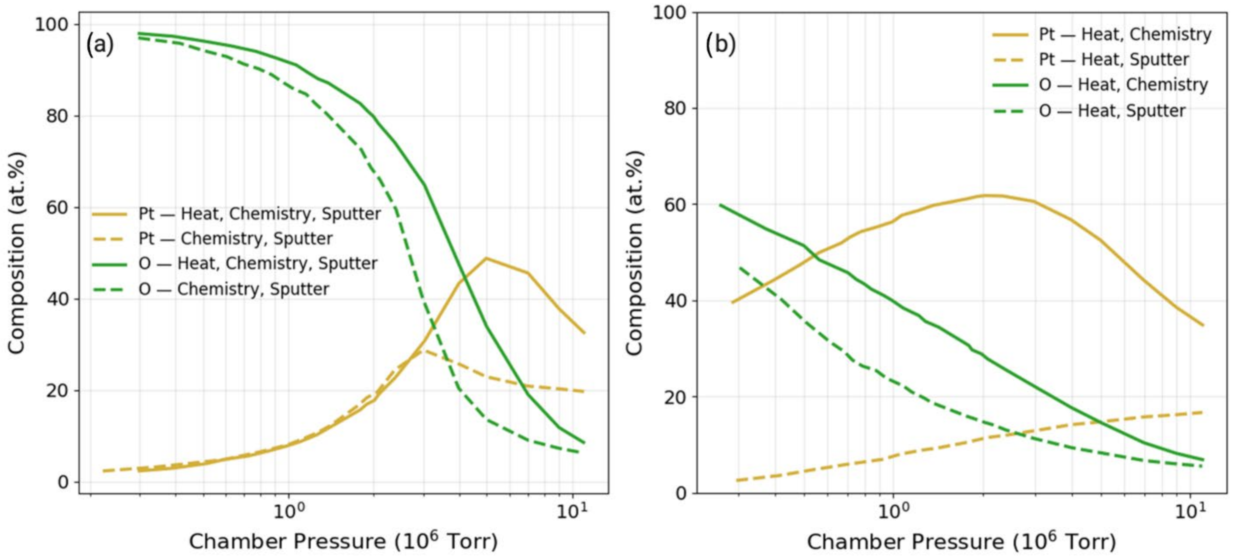

Figure S3: Sensitivity analysis on the numerical simulation with (a) all physics enabled, and beam heating disabled, and with (b) chemical etching and physical sputtering alone enabled. Legends indicate which of the 3 main physics modules are operative. Only platinum and oxygen content are shown for clarity as carbon is the remaining balance.

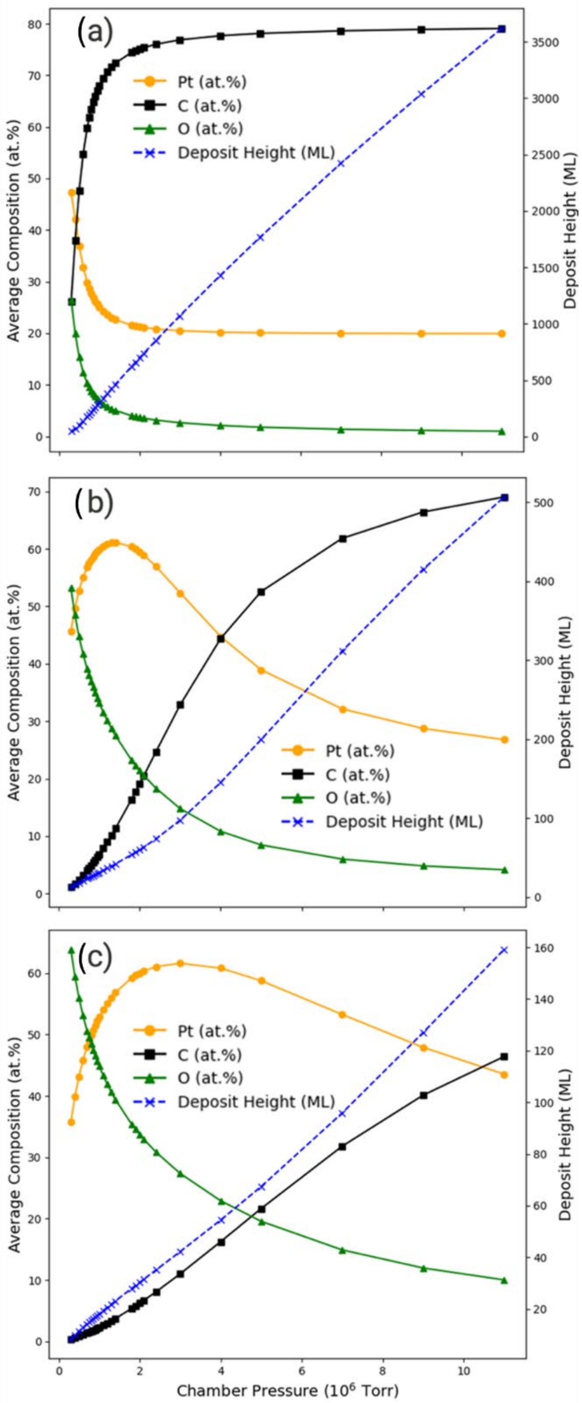

Figure S4: Sensitivity analysis of the numerical simulation with sputter yields of Pt and C inverted from those used throughout the work. Sputter yields used here were Pt = 0.08 and C = 0.02. Three pressure sweeps at dwell times of: (a) 500 ns, (b) 10 μs, and (c) 20 μs.

Maximum Pt values are higher in this case, but the general non-monotonic behavior is still observed.

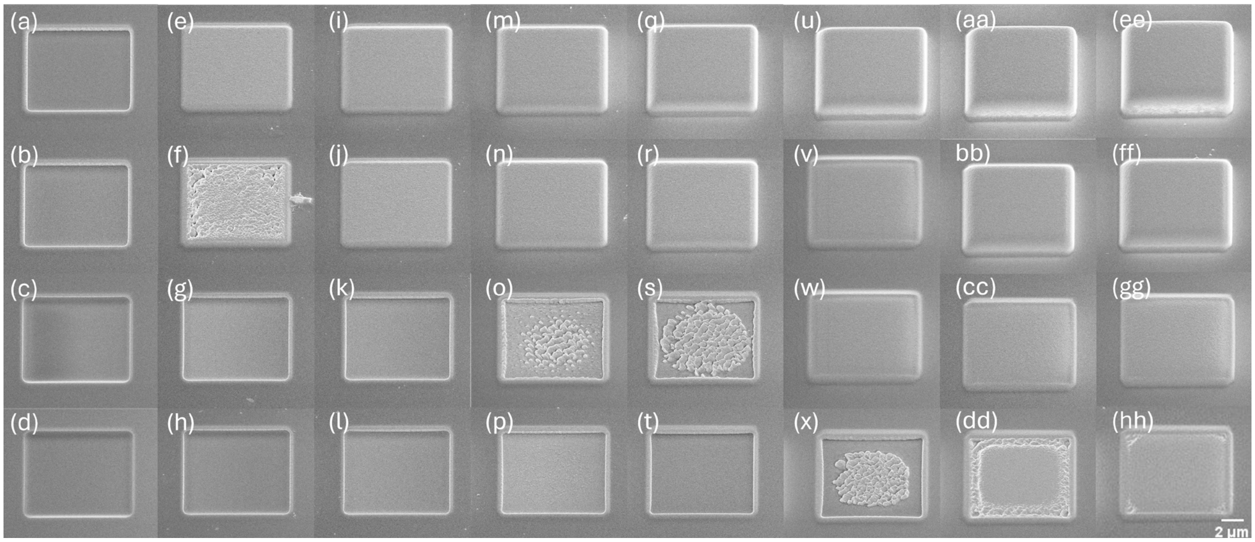

Figure S5: Top-down SEM micrographs of 10 x 10 μm square Pt deposits fabricated using a beam energy of 30 keV and beam current of 2.44 nA. Precursor depletion was systematically varied by adjusting the precursor duty cycle (a-d) 0.1, (e-h) 0.5, (i-l) 1, (m-p) 5, (q-t) 10, (u-x) 20, (aa-dd) 50, and (ee-hh) 95%. Within each duty-cycle group, the dwell time increases from top to bottom, corresponding to 0.15, 1, 10, and 20 μs, respectively. The scale bars are identical between images.

Table S2: Average platinum composition as a function of dwell time and duty cycle, reported as mean ± one standard deviation from triplicate depositions.

	Duty Cycle							
**Dwell Time (μs)**	**0.10%**	**0.50%**	**1%**	**5%**	**10%**	**20%**	**50%**	**95%**
**0.15**	7.9 ± 0.3	37.9 ± 0.3	32.7 ± 0.3	27.1 ± 0.2	25.9 ± 0.1	24.4 ± 0.5	24.0 ± 0.1	27.3 ± 6.2
**0.5**	7.8 ± 0.5	40.8 ± 0.5	34.5 ± 0.3	28.8 ± 0.1	27.5 ± 0.1	25.9 ± 0.7	25.2 ± 0.1	24.4 ± 0.9
**1**	8.2 ± 0.6	45.1 ± 0.3	38.7 ± 0.4	30.9 ± 0.2	29.1 ± 0.2	27.0 ± 0.9	25.9 ± 0.2	25.1 ± 0.8
**5**	7.2 ± 0.5	11.6 ± 0.2	13.4 ± 2.4	43.1 ± 1.2	35.1 ± 0.3	30.7 ± 0.9	28.3 ± 0.3	27.0 ± 0.4
**10**	6.1 ± 0.6	9.2 ± 0.0	10.1 ± 1.5	31.4 ± 16.4	42.8 ± 2.4	33.4 ± 2.2	31.3 ± 0.2	28.9 ± 0.7
**15**	5.3 ± 0.5	8.1 ± 0.1	9.1 ± 0.8	15.0 ± 3.0	33.5 ± 16.8	42.4 ± 5.0	34.1 ± 0.8	31.5 ± 0.7
**20**	5.1 ± 0.2	7.9 ± 0.3	8.6 ± 1.0	14.1 ± 2.8	26.7 ± 13.8	40.9 ± 2.5	39.7 ± 1.4	33.7 ± 2.3

Table S3: Corresponding average precursor pressure (chamber pressure minus base pressure) and chamber pressures associated with each duty cycle.

**Duty Cycle (%)**	**0.1**	**0.5**	**1**	**5**	**10**	**20**	**50**	**95**
Average Precursor Pressure (Torr)	4.29×10^{-9}	6.04×10^{-8}	1.18×10^{-7}	4.80×10^{-7}	8.67×10^{-7}	2.03×10^{-6}	2.51×10^{-6}	3.66×10^{-6}
Average Chamber Pressure (Torr)	1.15×10^{-6}	1.17×10^{-6}	1.41×10^{-6}	1.81×10^{-6}	2.25×10^{-6}	2.73×10^{-6}	4.08×10^{-6}	5.40×10^{-6}

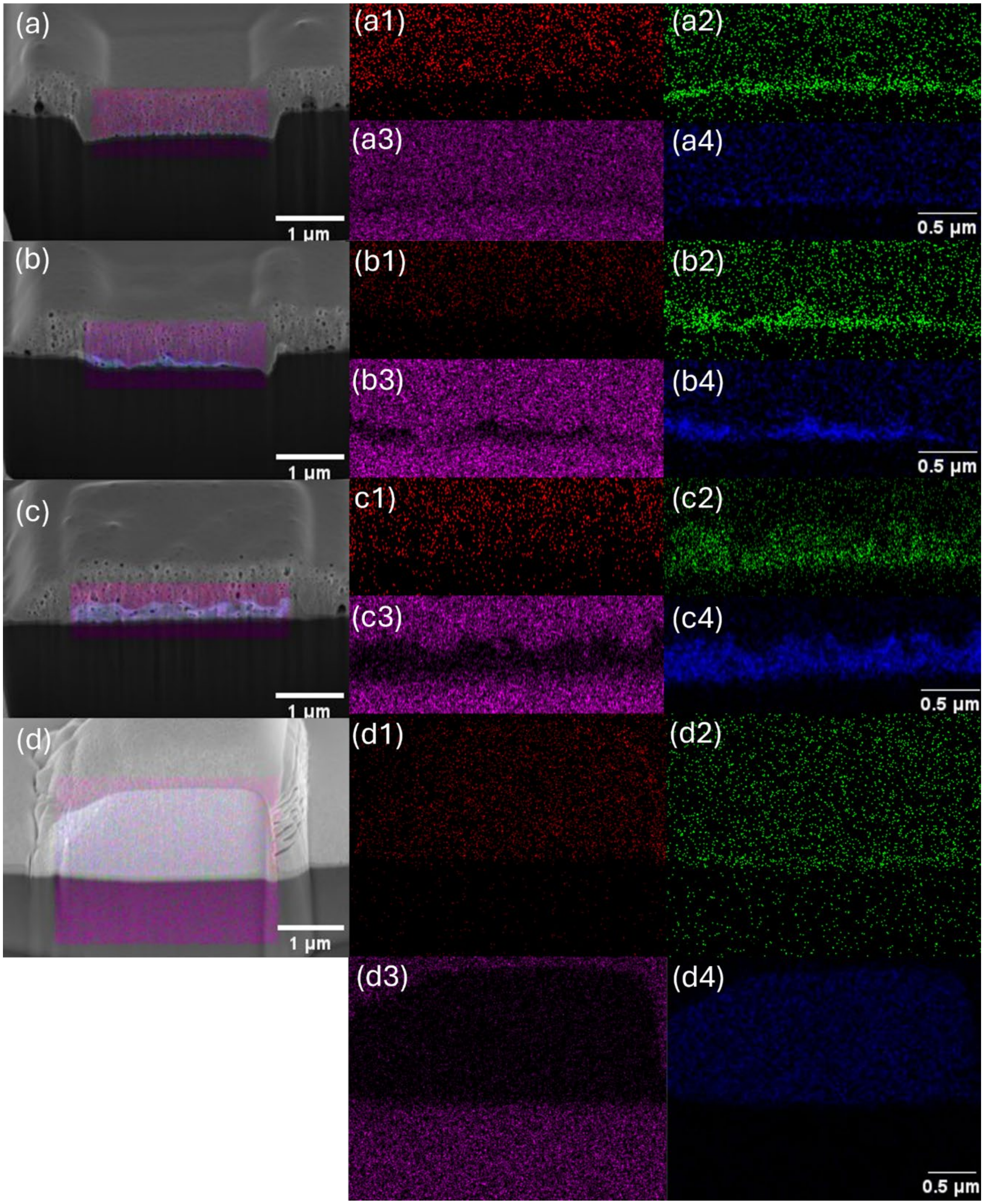

Figure S6: Cross-sectional SEM and EDS analysis of Pt deposits fabricated at 30 kV usingdwell times of (a) 1 µs, (b) 15 µs, (c) 35 µs, and (d) 150 µs. Panels (a–d) show cross-sectional SEM images overlaid with the combined EDS elemental maps. Corresponding individual elemental distributions are shown to the right for each dwell time: carbon (red: a1–d1), oxygen (green: a2–d2), tungsten (purple: a3–d3), and platinum (blue: a4–d4).
